# Curcumin in Combination With Omacetaxine Suppress Lymphoma Cell Growth, Migration, Invasion, and Angiogenesis *via* Inhibition of VEGF/Akt Signaling Pathway

**DOI:** 10.3389/fonc.2021.656045

**Published:** 2021-08-11

**Authors:** Yu Zhang, Jingjing Xiang, Ni Zhu, Hangping Ge, Xianfu Sheng, Shu Deng, Junfa Chen, Lihong Yu, Yan Zhou, Jianping Shen

**Affiliations:** ^1^Department of Hematology, First Affiliated Hospital of Zhejiang Chinese Medical University, Hangzhou, China; ^2^First Medical College, Zhejiang Chinese Medical University, Hangzhou, China

**Keywords:** lymphomas, homoharringtonine, curcumin, VEGF, exosomes

## Abstract

**Background:**

Both omacetaxine (HHT) and curcumin were shown to exhibit anti-proliferative effect on lymphoma cells. However, the role of combination of HHT with curcumin (HHT/curcumin combination) on lymphoma cells remains unclear. Thus, this study aimed to investigate the effect of HHT/curcumin combination on the proliferation, migration, and angiogenesis of lymphoma cells.

**Methods:**

Cell counting kit-8 (CCK-8), Ki67 immunofluorescence and transwell assays were used to assess the viability, proliferation and migration of U937 and Raji cells respectively. In addition, tube formation assay was used to determine the effects of HHT/curcumin combination on angiogenesis in human umbilical vein endothelial cells (HUVECs).

**Results:**

In this study, we found that HHT/curcumin combination significantly inhibited the proliferation, migration and invasion in U937 and Raji cells (all P < 0.01). In addition, combination treatment markedly inhibited the secreted levels of vascular endothelial growth factor (VEGF)-(A-D) (all P < 0.01) in Raji cells. Moreover, combination treatment exhibited anti-tumor effects in Raji cells, as shown by the decreased signals of phosphorylated VEGF receptor 2 (p-VEGFR2) and phosphorylated protein kinase B (p-Akt) (all P < 0.01). Meanwhile, combination treatment inhibited VEGFA levels (P < 0.01) in exosomes derived from Raji cells. Application of exosomes with downregulated VEGF to HUVECs notably inhibited proliferation, migration and tube formation of HUVECs, evidenced by the decreased signals of p-Akt, angiogenin-1, matrix metallopeptidase 2 (MMP2) and matrix metallopeptidase 9 (MMP9) (all P < 0.01).

**Conclusion:**

Our findings indicated that combination of HHT and curcumin could inhibit lymphoma cell growth and angiogenesis *via* inhibition of VEGF/Akt signaling pathway. These results suggested that HHT combined with curcumin might be regarded as a promising therapeutic approach for the treatment of lymphoma.

## Introduction

Lymphomas are a heterogeneous group of lymphoid malignancies that derives from lymph gland and/or extranodal lymphoid tissue (1, 2). Lymphomas can be categorized into two main types: Hodgkin’s Disease (HD) and Non‐Hodgkin’s Lymphoma (NHL) according to the World Health Organization (WHO) classification (2, 3). In addition, NHL can be further divided into neoplasms of B-cell, T-cell or NK/T cell origin (1, 4). Burkitt’s lymphoma (BL) is a highly invasive B-cell NHL characterized by a high proliferation rate of the malignant cells (5, 6). Evidence has shown that BL is highly sensitive to chemotherapy (7). Chemotherapy is effective in the treatment of BL but associated with unavoidable toxicity and side effects (8). Therefore, novel strategies for the treatment of lymphomas are imminently needed.

Omacetaxine, formerly known as homoharringtonine (HHT), is a natural alkaloid derived from Cephalotoxus fortunei with antitumor properties (9, 10). US Food and Drug Administration (FDA) approved HHT for the treatment chronic myeloid leukemia (11). Nguyen et al. indicated that combination of HHT and bortezomib could inhibit the growth of diffuse large B-cell lymphoma (DLBCL) cells (12). Curcumin is a polyphenol derived from the dried rhizome of *Curcuma longa* L (13). Curcumin has extensive biological activities such as anti-tumor, anti-inflammatory, anti-angiogenesis (14–16). Chen et al. found that curcumin could suppress the proliferation and invasion of DLBCL cells (17). It has been shown that combination of anti-cancer drugs in cancer therapies have the potential of improving the effectiveness of drug treatment (18). Therefore, this study aimed to investigate the anti-tumor effects of HHT/curcumin combination in lymphoma cells.

Exosomes are extracellular vesicles ranging from 50 to 150 nm in size that contain proteins, lipids, mRNAs and miRNAs (19). Exosomes play important roles in remodeling the extracellular environment and regulating intercellular communication (20). It has been shown that tumor cells can secrete many more exosomes than normal cells (21). Cancer cell-derived exosomes can delivery genetic material to recipient cells in tumor environment (22, 23). Meanwhile, tumor-derived exosomes were shown to promote angiogenesis in endothelial cells (24). However, whether HHT and curcumin can affect the secretion of lymphoma exosomes remains unclear. Raji cells are tumor cells that originate from human BL (25). In addition, human lymphoma cell line U937 was derived from a patient with generalized histiocytic lymphoma, which has been used extensively for studying the induction of apoptosis and differentiation (26, 27). Therefore, this study aimed to investigate the anti-tumor effects of HHT/curcumin combination in Raji and U937 cells. Meanwhile, we sought to determine whether HHT and curcumin can affect the secretion of lymphoma exosomes thereby suppressing tumor angiogenesis.

## Materials And Methods

### Cell Culture

Human umbilical vein endothelial cells (HUVECs), human leukemic monocyte lymphoma cell line U937, Burkitt’s lymphoma cell line Raji and human bone marrow stromal cell line HS-5 were purchased form American Type Culture Collection (ATCC, Rockville, Maryland, USA). Cells were cultured in high-content glucose DMEM medium (cat. no. 11965092; Thermo Fisher Scientific, Waltham, MA, USA) supplemented with 10% FBS (cat. no. 10091148; Thermo Fisher Scientific), 1% penicillin-streptomycin (cat. no. 15070063; Thermo Fisher Scientific). Cells were incubated in a humidified incubator containing 5% CO_2_ at 37°C.

### Cell Counting Kit-8 Assay

U937 and Raji cells (5000 cells/well) were seed onto 96-well plates and incubated overnight. After that, U937 and Raji cells were treated with 5 ng/mL HHT or/and 10 μM curcumin for 72 h. Later on, 10 μL CCK‐8 reagent (cat. no. C0038; Beyotime) was added into each well, and cells were incubated for another 2 h. Subsequently, the absorbance at a wavelength of 450 nm was measured using the Multiskan™ FC Microplate Photometer (Thermo Fisher Scientific).

### Immunofluorescence Assay

Cells were fixed with 4% paraformaldehyde (cat. no. AS1018; ASPEN Biotechnology) for 15 min, and then incubated with 0.1% Triton X-100 (cat. no. ST797; Beyotime) for 5 min. Later on, cells were blocked with 1% BSA (cat. no. 10735078001; Roche) for 30 min, followed by incubation with the Ki67 antibody (cat. no. ab92742; Abcam Cambridge, MA, USA) overnight at 4°C. After that, the cells were incubated with a secondary antibody (cat. no. ab150077; Abcam) at 37°C for 1 h. Nuclei were counterstained with DAPI (100 μl; cat. no. C1005, Beyotime, Shanghai, China). Subsequently, fluorescence signals were imaged with a fluorescence microscope (Carl Zeiss, Jena, Germany).

### Transwell Assays

Transwell migration and invasion assays were performed using the Matrigel-uncoated or Matrigel-coated transwell chambers (8 μm; Corning, USA) respectively. U937 and Raji cells (4 x 10^4^ cells/well) were suspended in 200 μL serum-free culture medium and then seed into the upper chamber, and 700 μL of DMEM medium containing 2% FBS was added into the lower chamber. Twenty-four hours later, cells that migrated or invaded through the transwell membrane were fixed with 70% methanol, and then stained with 1% crystal violet (cat. no. AS1086; ASPEN Biotechnology). Subsequently, the migrated or invaded cells were observed using a fluorescence microscope and counted in five randomly selected fields.

### ELISA

The samples of the supernatant were collected from Raji cells. After that, ELISA kits for VEGF-A (cat. no. ELK1129), VEGF-B (cat. no. ELK2321), VEGF-C (cat. no. ELK1194), VEGF-D (cat. no. ELK1195) were purchased from ELK Biotechnonlgy (Wuhan, China), and the assay was performed according to the manufacturer’s protocols. Absorbance was assessed using a Multiskan™ FC Microplate Photometer (Thermo Fisher Scientific).

### RT-qPCR

Total RNA was extract using the TRIpure Total RNA Extraction Reagent (cat. no. EP013; ELK Biotechnology, Wuhan, China). After that, cDNA was synthesized using EntiLink™ 1st Strand cDNA Synthesis Kit (cat. no. EQ003; ELK Biotechnology). Later on, qPCR was performed on the StepOne™ Real-Time PCR System (Thermo Fisher Scientific) using the EnTurbo™ SYBR Green PCR SuperMix (cat. no. EQ001; ELK Biotechnology). Expression of target genes (2^−ΔΔCt^) was normalized against β-actin and U6, as described previously (28). U6: 5’-CTCGCTTCGGCAGCACAT-3’ (F); 5’- AACGCTTCACGAATTTGCGT-3’ (R). miR-150: 5’- TCCCAACCCTTGTACCAGTG-3’ (F); 5’-CTCAACTGGTGTCGTGGAGTC-3’ (R). miR-22: 5’-GCCAGTTGAAGAACTGTCTCAAC-3’ (F); 5’- CTCAACTGGTGTCGTGGAGTC-3’ (R). miR-206: 5’- TGGAATGTAAGGAAGTGTGTGG-3’ (F); 5’-CTCAACTGGTGTCGTGGAGTC-3’ (R). miR-34a: 5’-TGGCAGTGTCTTAGCTGGTTG-3’ (F); 5’- CTCAACTGGTGTCGTGGAGTC-3’ (R). β-actin: 5’- GTCCACCGCAAATGCTTCTA-3’ (F); 5’-TGCTGTCACCTTCACCGTTC-3’ (R). VEGFA: 5’-GAACTTTCTGCTGTCTTGGGTG-3’ (F); 5’- GGCAGTAGCTGCGCTGATAG-3’ (R).

### Western Blot Assay

Total proteins were quantified using Bradford protein assay (Bio-Rad, CA, USA), and then equal proteins (30 μg per lane) were separated by 10% SDS-PAGE (cat. no. AS1012; ASPEN). After that, proteins were transferred onto polyvinylidene difluoride (PVDF) membrane (Thermo Fisher Scientific). Later on, the membrane was blocked in 5% skimmed milk (cat. no. AS1033; ASPEN) for 1 h at room temperature, followed by incubating with the primary antibodies against p-VEGFR2 (cat. no. ab5473; 1:1000, Abcam), VEGFR2 (cat. no. ab134191; 1:1000, Abcam), p-Akt (cat. no. ab81283; 1:1000, Abcam), Akt (cat. no. ab179463; 1:1000, Abcam), p-eNOS (cat. no. ab215717; 1:1000, Abcam), eNOS (cat. no. ab252439; 1:1000, Abcam), p-p38 (cat. no. #4631; 1:1000, Cell signaling technology), p38 (cat. no. #8690, 1:1000, Cell signaling technology), p-HSP27 (cat. no. #9709; 1:1000, Cell signaling technology), HSP37 (cat. no. #95357; 1:1000, Cell signaling technology), p-ERK (cat. no. ab201015; 1:1000, Abcam), ERK (cat. no. ab184699; 1:1000, Abcam), p-JNK (cat. no. ab112501; 1:1000, Abcam), JNK (cat. no. ab4821; 1:1000, Abcam), VEGFA (cat. no. ab52917; 1:1000, Abcam), angiogenin-1 (cat. no. ab189207; 1:1000, Abcam), MMP2 (cat. no. ab92536; 1:1000, Abcam), MMP9 (cat. no. ab76003; 1:1000, Abcam) p-STAT3 (cat. no. ab267373; 1:1000, Abcam), STAT3 (cat. no. ab68153; 1:1000, Abcam), SP1 (cat. no. ab124804; 1:1000, Abcam) and anti-β-actin (cat. no. ab8227; 1:1000) at 4°C overnight. Next, the membrane was incubated with horseradish peroxidase-conjugated secondary antibodies (cat. no. ab97051; 1: 5000) for 1 h. Subsequently, the blots were visualized using an electrochemiluminescence (Thermo Fisher Scientific). β-actin was acted as an internal control.

### Flow Cytometry Assay

Cell apoptosis were carried out using the fluorescein isothiocyanate (FITC) Annexin V Apoptosis Detection Kit (cat. no. 559763; BD Biosciences). A FACSCalibur flow cytometer (BD Biosciences) was used to analyze the number of apoptotic cells.

### Isolation and Characterization of Exosomes

Exosomes were purified from Raji cell-derived conditioned media (CM) and mouse serum by ultracentrifugation, following previously described standard procedures (29). The exosomes pellets were washed with PBS followed by ultracentrifugation at 100,000*g* at 4°C for 60 min, and then suspended in PBS for further processed for protein or RNA extraction. The nanoparticle tracking analysis (NTA; Malvern Panalytical, Malvern, UK) was used to assess the size and quantity of exosomes. Western blot assay was performed to detect exosome surface markers (TSG101 and CD63).

### Co-Culture of Exosomes and HUVECs

Exosomes isolated from Raji cells were fluorescently labeled with PKH26 membrane dye (cat. no. MINI26; Sigma Aldrich, St. Louis, MO, USA) and incubated for 15 min at 37°C. Later on, labeled exosomes were washed exosome-free PBS by ultracentrifugation at 110,000*g*, and then resuspended in exosome-free PBS. After that, PKH26-traced exosomes were co-cultured with HUVECs for 24 h. F-actin was stained using phalloidin-FITC. Subsequently, internalization of exosomes by HUVECs were observed by a fluorescence microscope.

### Tube Formation Assay

100 μL growth factor enriched Matrigel (cat. no. 354248; Corning) was plated in 24-well plate and allowed to polymerize for 30 min at 37°C. After that, the treated HUVECs were resuspended in serum-free medium and seeded on the matrigel-coated well. The tube formation of HUVECs was observed and photographed after 12 h incubation under a microscope. The tube formation ability was determined by measuring the branch points, mesh area and tube numbers with five randomly selected fields.

### Animal Study

BALB/c nude mice (6 – 8 weeks old) were obtained from the Shanghai Slac Animal Center (Shanghai, China). All animal experiments were approved by the First Affiliated Hospital of Zhejiang Chinese Medical University and animals were maintained following the recommended procedures of the National Institutes of Health Guide for the Care and Use of laboratory animals. Raji cells (5 x 10^6^ cells) were injected subcutaneously into the left flank of nude mice. When the tumors volume reached to 180 mm^3^, animals were randomized into two groups: control and Exo^-HHT + Curcumin^ groups. The control group received normal saline only. In addition, mice in the Exo^-HHT + Curcumin^ group were intravenously injected with Exo^-HHT + Curcumin^ every 2 days. Tumor volume was obtained by calipers once a week. After that, animals were sacrificed under anesthesia at day 28 and the tumors were removed.

### Statistical Analysis

All statistical analyses were performed using GraphPad Prism software (version 7.0, La Jolla, CA, USA). The normality of the data was tested using Shapiro-Wilk test. One-way analysis of variance (ANOVA) and Tukey’s tests were carried out for multiple group comparisons. Each experiment was carried out in triplicate. Data are presented as the mean ± standard deviation (S.D.). *P < 0.05 was considered to be statistically significant.

## Results

### Combination of Curcumin With HHT Inhibited the Proliferation of Lymphoma Cells

**Figure 1:**
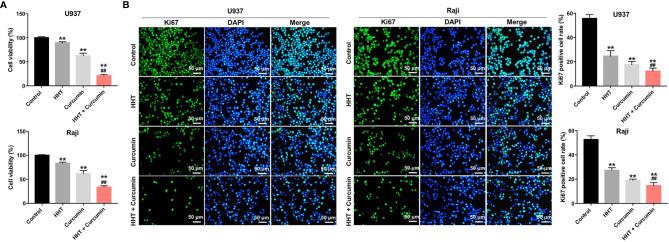
Combination of curcumin with HHT inhibited the proliferation of lymphoma cells. U937 and Raji cells were treated with 5 ng/mL HHT or/and 10 μM curcumin for 72 h. **(A)** CCK-8 assay was used to detect cell viability. **(B)** Relative fluorescence expression levels were quantified by Ki67 and DAPI staining. **P < 0.01 compared with control group; ^##^P < 0.01 compared with HHT group.

To explore the role of curcumin and HHT on the viability of lymphoma cells, CCK-8 assay was performed. As shown in **Figure 1A**, treatment of both U937 and Raji cells with HHT or curcumin alone significantly decreased the viability compared with the control group (P < 0.01). Remarkably, combination treatment with HHT and curcumin reduced cell viability more in U937 and Raji cells than either HHT or curcumin alone (P < 0.01; **Figure 1A**). In addition, the results of ki67 immunofluorescence assay indicated that HHT treatment notably inhibited the proliferation of U937 and Raji cells (P < 0.01; **Figure 1B**). As expected, combined treatment with curcumin potentiated the anti-proliferative effects of HHT on U937 and Raji cells (P < 0.01; **Figure 1B**). These data suggested that combination of curcumin with HHT could inhibit the proliferation of lymphoma cells.

### Combination of Curcumin With HHT Suppressed the Migration and Invasion Abilities of Lymphoma Cells

**Figure 2:**
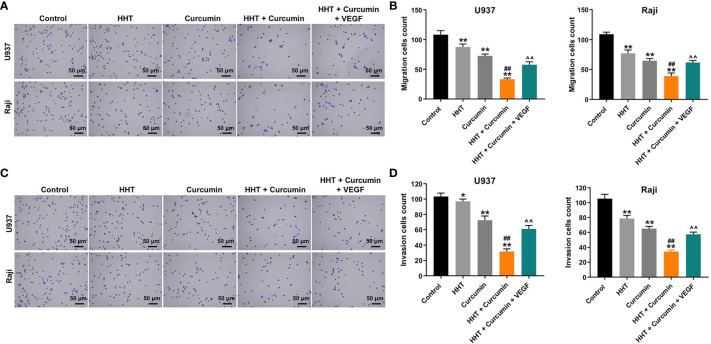
Combination of curcumin with HHT suppressed the migration and invasion abilities of lymphoma cells. U937 and Raji cells were treated with 5 ng/mL HHT or/and 10 μM curcumin, or treated with HHT, curcumin and VEGF for 24 h. **(A, B)** Cell migration was detected by transwell migration assay. **(C, D)** Cell invasion was detected by transwell invasion assay. *P < 0.05, **P < 0.01 compared with control group; ^##^P < 0.01 compared with HHT group; ^^^^P < 0.01 compared with HHT + curcumin group.

Next, the effects of HHT/curcumin combination on cell migration and invasion were detected with transwell assay. As shown in **Figures 2A–D**, combination treatment significantly inhibited the migration and invasion abilities of U937 and Raji cells; however, these changes were obviously reversed by VEGF treatment (All P < 0.01). These results indicated that combination of curcumin with HHT could suppress the migration and invasion abilities of lymphoma cells.

### Combination of Curcumin With HHT Inhibited The Growth of Lymphoma Cells *via* Downregulation of VEGF

**Figure 3:**
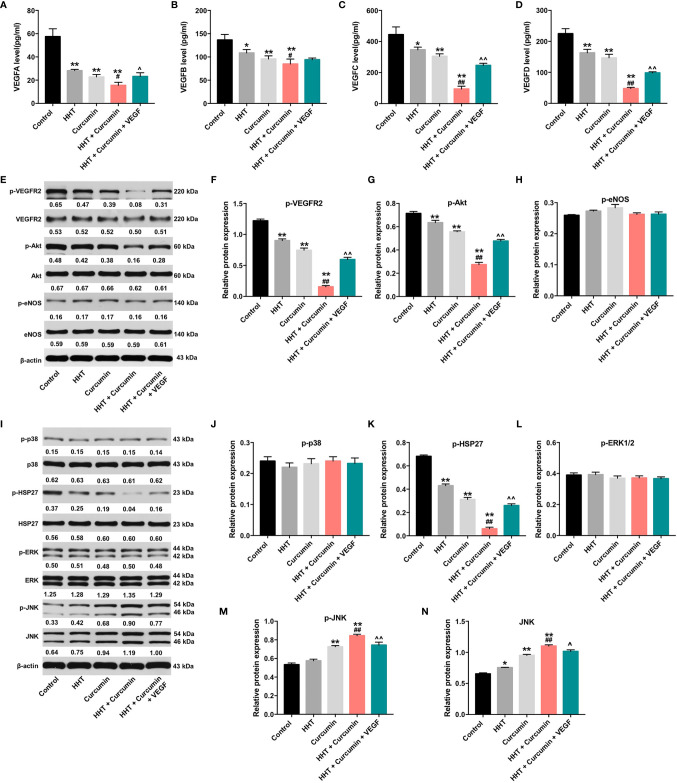
Combination of curcumin with HHT inhibit the growth of lymphoma cells *via* inhibition of VEGF/VEGFR2 signaling pathway. Raji cells were treated with 5 ng/mL HHT or/and 10 μM curcumin for 72 h or treated with HHT, curcumin and VEGF. The levels of **(A)** VEGF-A, **(B)** VEGF-B, **(C)** VEGF-C, **(D)** VEGF-D in cell culture supernatant was assessed with ELISA. **(E)** Expression levels of p-VEGFR2, VEGFR2, p-Akt, Akt, p-eNOS and eNOS in Raji cells were detected with western blotting. **(E–H)** The relative expressions of p-VEGFR2, p-Akt, and p-eNOS in cells were quantified *via* normalization to VEGFR2, Akt and eNOS. **(I)** Expression levels of p-p38, p38, p-HSP27, HSP27, p-ERK, ERK, p-JNK, JNK in Raji cells were detected with western blotting. **(J–N)** The relative expressions of p-p38, p-HSP27, p-ERK, p-JNK, JNK in cells were quantified *via* normalization to p38, HSP27, ERK, JNK and β-actin. *P < 0.05, **P < 0.01 compared with control group; ^#^P < 0.05, ^##^P < 0.01 compared with HHT group; ^^^P < 0.05, ^^^^P < 0.01 compared with HHT + curcumin group.

VEGF is released by cancer cells to induce tumor growth, migration and angiogenesis (30). ELISA assay was used to detect the secreted levels of VEGF-A, VEGF-B, VEGF-C and VEGF-D in Raji cells. As shown in **Figures 3A–D**, combination of HHT with curcumin decreased the secreted levels of VEGFA (P < 0.05), VEGFB (P < 0.05), VEGFC (P < 0.01) and VEGFD (P < 0.01) in Raji cells compared with HHT treatment group; however, these effects were reversed by VEGF treatment. To further explore whether combination treatment inhibited the viability and migration *via* regulation of VEGFA, we used two siRNAs to downregulate the level of VEGFA in Raji cells (**Supplementary Figure 1A**). Additionally, VEGFA siRNAs enhanced the inhibitory effects of combination treatment on the viability (P < 0.01) and migration (P < 0.01) of Raji cells (**Supplementary Figures 1B, C**). These data suggested that combination treatment could inhibit the viability and migration of lymphoma cells *via* downregulation of VEGFA.

In addition, it has been shown that Sp1 and STAT3 are transcription factors that regulate VEGF expression and VEGF secretion of cancer cells (31, 32). As shown in **Supplementary Figures 2A, B**, combination of HHT with curcumin remarkedly decreased the expressions of p-STAT3 and SP1 in Raji cells; however, these effects were reversed by VEGF treatment (All P < 0.01). These results indicated HHT/curcumin combination inhibited VEGF level in Raji cells *via* inactivation of the transcription factors STAT3 and SP1.

Moreover, evidences have shown that VEGFA expression can be regulated by various microRNAs (miRNAs), including of miR-150, miR-22, miR-206 and miR-34a (33–36). Meanwhile, the levels of miR-150, miR-22, miR-206 and miR-34a have been found to be downregulated in human cancers (37–40). Thus, we investigated whether HHT combined with curcumin reduced VEGFA level in Raji cells by these miRNAs. As shown in **Supplementary Figure 3**, combination treatment significantly upregulated the levels of miR-150 (P < 0.01), miR-22 (P < 0.01), miR-206 (P < 0.05) and miR-34a (P < 0.01) in Raji cells. Collectively, HHT/curcumin combination decreased VEGFA level in Raji cells *via* upregulation of miR-150, miR-22, miR-206 and miR-34a.

### Combination of Curcumin With HHT Inhibited the Growth of Lymphoma Cells *via* Regulation of VEGF/Akt and JNK Signaling Pathways

It has been reported that PI3K/Akt and MAPK signaling pathways could be mediated by VEGF (41, 42). Thus, we next to examine whether HHT/curcumin combination inhibited Raji cell growth *via* regulation of PI3K/Akt and MAPK signaling pathways (**Figures 3E–N**). As indicated in **Figures 3E–G, I, K, M, N**, combination treatment led to decreased phosphorylation levels of VEGFR2 (P < 0.01), Akt (P < 0.01), p-HSP27 (P < 0.01) proteins and increased phosphorylated and total JNK (P < 0.01) levels in Raji cells; however, these levels were reversed in the presence of VEGF. Meanwhile, combination treatment caused no difference in the activation of eNOS, p38 and ERK1/2 in Raji cells (**Figures 3E, H, I, J, L**). These data illustrated that combination of curcumin with HHT could inhibit the growth of lymphoma cells *via* regulation of VEGF/Akt and JNK signaling pathways.

### Combination of Curcumin With HHT Inhibited VEGF Levels in Exosomes Derived From Raji Cells

**Figure 4:**
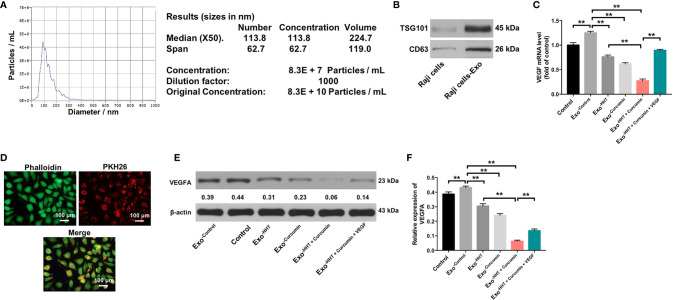
Lymphomas exosomes are absorbed by HUVECs. **(A)** The mean diameter of lymphomas exosomes was analyzed using the NTA. **(B)** Western blot analysis of the exosome marker TSG101 and CD63 in Raji cells and the exosomes isolated from Raji cells. **(C)** RT-qPCR analysis shows VEGF levels in exosomes obtained from Raji cells that were treated with HHT or/and curcumin or treated with HHT, curcumin and VEGF. **(D)** Confocal microscopy image shows presence of PKH26 dye (Red) in HUVECs after adding PKH26-labeled exosomes derived from Raji cells. Phalloidin (green) was used to stain F-actin. **(E, F)** HUVECs were co-cultured with exosomes obtained from Raji cells that were treated with HHT or/and curcumin or treated with HHT, curcumin and VEGF. Expression level of VEGFA in HUVECs were detected with western blotting. **P < 0.01.

It has been shown that cancer-secreted exosomes are responsible for tumor growth and angiogenesis (43). In order to explore whether lymphoma cell-secreted exosomes perform the above functions, we extracted exosomes from the cultural supernatants of Raji cells. Nanoparticle tracking analysis (NTA) showed that the lymphomas exosomes were approximately 50-150 nm in diameter (**Figure 4A**). In addition, western blot revealed that vesicles were positive for exosome markers TSG101 and CD63 (**Figure 4B**). RT-qPCR showed that exosomal VEGF was found to be enriched in the conditioned medium of Raji cells (P < 0.01). However, Raji cells treated with HHT and curcumin led to the downregulation of exosomal VEGF; whereas additional of exogenous VEGF reversed the effect of HHT/curcumin combination on the level of VEGF in lymphoma cell-secreted exosomes (All P < 0.01; **Figure 4C**). These data indicated that combination of curcumin with HHT could inhibit VEGF levels in exosomes derived from Raji cells.

### Lymphomas Exosomes Are Absorbed by HUVECs

Next, Raji cell derived PKH26-labeled exosomes were incubated with HUVECs. After 24 h of co-cultivation, PKH26-labeled dye was observed in HUVECs (**Figure 4D**). These results suggested that lymphomas exosomes are absorbed by HUVECs. We further detected the expression of VEGF in HUVECs incubated with exosomes isolated from Raji cells. As shown in **Figures 4E, F**, the expression of VEGF was markedly upregulated in HUVECs incubated with lymphoma cell-secreted exosomes; however, VEGF expression in HUVECs was markedly downregulated by the exosomes isolated from HHT/curcumin combination-treated Raji cells (Raji/combination exosomes) compared to those isolated from control Raji cells (All P < 0.01). These results suggested that HHT combined with curcumin treatment reduced VEGF secretion from Raji cells.

### Raji/Combination Exosomes Inhibited Proliferation and Migration of HUVECs *via* Reducing VEGF Secretion From Raji Cells

**Figure 5:**
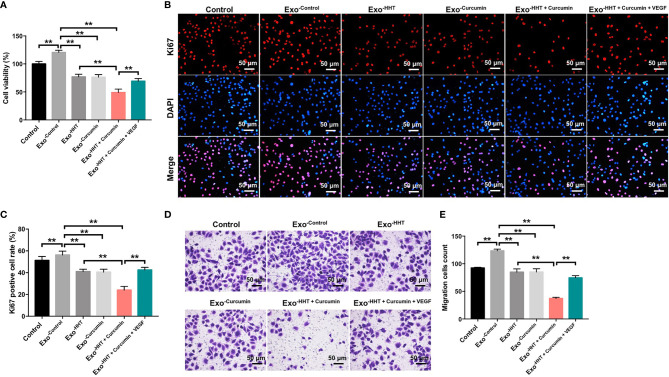
Raji/combination exosomes inhibits proliferation and migration of HUVECs. HUVECs were co-cultured with exosomes obtained from Raji cells that were treated with HHT or/and curcumin or treated with HHT, curcumin and VEGF. **(A)** CCK-8 assay was used to detect cell viability. **(B, C)** Relative fluorescence expression levels were quantified by Ki67 and DAPI staining. **(D, E)** Cell migration was detected by transwell migration assay. **P < 0.01.

To further explore whether Raji/combination exosomes could inhibit the proliferation and migration of HUVECs, CCK-8, Ki67 immunofluorescence and transwell migration assays were applied. As shown in **Figures 5A–E**, Raji cell-derived exosomes significantly promoted the viability, proliferation and migration of HUVECs (All P < 0.01). In contrast, the viability, proliferation and migration of HUVECs were markedly inhibited by the Raji/combination exosomes compared to those purified from control Raji cells; however, these changes were reversed by the exosomes loaded with exogenous VEGF (All P < 0.01; **Figures 5A–E**). These results indicated that Raji/combination exosomes could inhibit proliferation and migration of HUVECs by reducing VEGF secretion from Raji cells.

### Raji/Combination Exosomes Inhibited Angiogenesis of HUVECs *via* Reducing VEGF Secretion From Raji Cells

**Figure 6:**
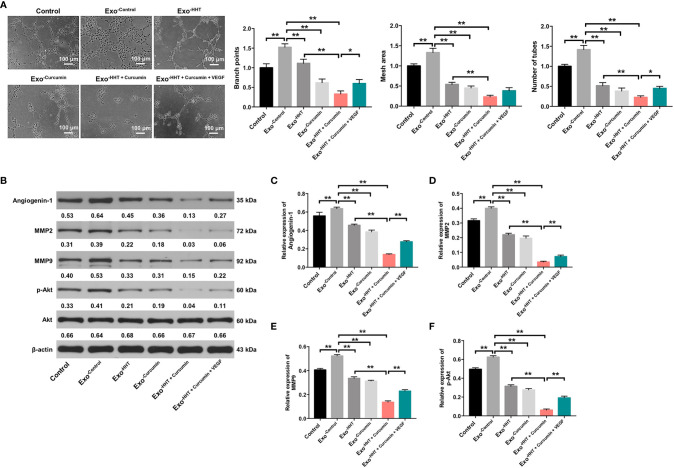
Raji/combination exosomes inhibits the angiogenesis of HUVECs. HUVECs were co-cultured with exosomes obtained from Raji cells that were treated with HHT or/and curcumin or treated with HHT, curcumin and VEGF. **(A)** The tube formation ability of HUVECs was detected by tube formation assay. Scale bar, 100 μm. **(B)** Expression levels of Angiogenin-1, MMP2, MMP9 and p-Akt in HUVECs were detected with western blotting. **(C–F)** The relative expressions of Angiogenin-1, MMP2 and MMP9 in cells were quantified *via* normalization to β-actin. The relative expression of p-Akt in cells were quantified *via* normalization to Akt. *P < 0.05, **P < 0.01.

Tube formation assay were then performed to assess whether Raji/combination exosomes could regulate angiogenesis in HUVECs. As indicated in **Figure 6A**, exosomes from Raji cells notably promoted angiogenesis in HUVECs, as shown by branch points (+52%; P < 0.01), mesh area (+33%; P < 0.01) and tube number (+42%; P < 0.01) increases. On the contrary, Raji/combination exosomes dampened angiogenesis in Raji cells (P < 0.01; **Figure 6A**). In addition, western blot assay indicated that the expressions of angiogenin-1, MMP2, MMP9 and p-Akt were obviously decreased in HUVECs by the Raji/combination exosomes compared to those purified from control Raji cells; however, these levels were reversed by the exosomes loaded with exogenous VEGF (All P < 0.01; **Figures 6B–F**). These data indicated that Raji/combination exosomes could inhibit the angiogenesis of HUVECs by reducing VEGF secretion from Raji cells.

Additionally, stromal cells have been characterized as important component of the tumor microenvironment as well, which play an important role in cancer development (44). Meanwhile, adhesion to cultured stromal cells could protect lymphoma cells from apoptosis induced by anti-tumor drugs (45). Thus, we established HS-5-Raji co-culture model in direct contact mode to simulate the bone marrow microenvironment in lymphoma (45). As shown in **Supplementary Figure 4B**, the inhibitory effect of combination treatment on cell viability was reversed by co-culturing of Raji cells with HS-5 (P<0.01). In addition, co-cultures of Raji cells with HS-5 led to a marked decrease of combination treatment-induced apoptosis compared to cells cultured on a plastic surface (17% vs. 25%, P<0.01). Collectively, interactions with the microenvironment could protect Raji cells from combination treatment-induced apoptosis. Again, these results proved that interactions with the microenvironment might be critical for mediating tumor therapy.

### Raji/Combination Exosomes Inhibited Tumorigenesis in Raji Xenograft *In Vivo*

**Figure 7:**
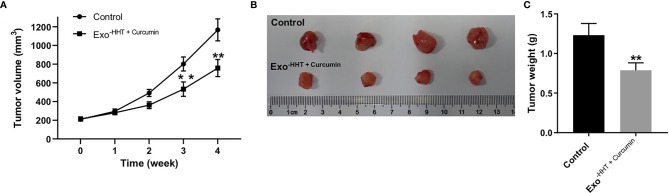
Raji/combination exosomes inhibited tumorigenesis in Raji xenograft *in vivo*. **(A)** Tumor volume was calculated. **(B)** Raji xenograft tumors were photographed, and **(C)** the tumor tissues were weighed. **P < 0.01 compared with control group.

Next, we investigated the anti-tumor effect of Raji/combination exosomes in mouse Raji xenograft model *in vivo*. As shown in **Figures 7A–C**, Raji/combination exosomes markedly reduced the tumor volume (P < 0.01) and tumor weight (P < 0.01) of Raji transplanted tumor *in vivo*. Collectively, Raji/combination exosomes could inhibit tumorigenesis in Raji xenograft *in vivo*.

## Discussion

In this study, we found that combination of curcumin with HHT could inhibit the proliferation of lymphoma cells. Moreover, combination of curcumin with HHT suppressed the migration and invasion *via* inhibition of VEGF signaling. Meanwhile, Raji/combination exosomes could inhibit proliferation, migration and angiogenesis of HUVECs *via* reducing VEGF secretion from Raji cells.

Evidence has been shown that VEGF correlated with angiogenesis and tumor growth in NHLs and HLs (46, 47). VEGF family members have already known to stimulate angiogenesis and enable tumor neovascularization (48, 49). Hoeres et al. found that lymphoma cell lines could produce VEGF, as well as release soluble VEGFR‐1 simultaneously (50). Meanwhile, zhang et al. indicated that curcumin could suppress the lymphangiogenesis in gastric cancer cells *via* inhibition of VEGFD (51). In this study, we found that combination of HHT with curcumin significantly decreased the secreted levels of VEGF-(A-D) in Raji cells, as well as reduced phosphorylation of VEGFR2 protein. These data indicated that combination treatment could suppress the VEGF signaling in Raji cells.

It has been shown that VEGF binds to VEGFR2, and activates the downstream targets of the PI3K/Akt, ERK1/2 and p38 MAPK pathways (52–55). Meanwhile, VEGF administration also suppressed the sustained activation of JNK by serum starvation (55). In this study, we found that combination treatment caused decreased phosphorylation level of Akt in Raji cells, but, that phenomenon was reversed by VEGF treatment. Meng et al. found that HHT could induce the apoptosis of multiple myeloma cells *via* suppression of the Akt pathway, which was consistent with our results (56). The results indicated that combination treatment exhibited anti-tumor effects in Raji cells through inhibition of VEGF/AKT signaling.

In addition, our data found that combination treatment caused no difference in the activation of p38 and ERK1/2 in Raji cells, while combination treatment significantly increased the phosphorylation level of JNK in Raji cells. He et al. found that H_2_O_2_ treatment significantly induced apoptosis of endothelial cells, and induced a transient activation of ERK1/2, p38, and JNK in a short time (<2 h), while no differences in the phosphorylation of ERK1/2 and p38 were detected when cells cultured with H_2_O_2_ for 24 h (57). Interestingly, H_2_O_2_ led to a persistent activation of JNK in endothelial cells, and VEGF administration could inhibit H_2_O_2_-induced apoptosis and suppress the activation of JNK, which were consistent with our results (57). Naserian et al. reported that combination of metformin with dacarbazine notably induced the apoptosis in Raji cells *via* activation of JNK signaling (58). Prolonged JNK activation contributes to cell apoptosis (59). The results indicated that combination treatment exhibited anti-tumor effects in Raji cells through inhibition of VEGF, and then activating JNK signaling.

VEGF has been reported to be responsible for the communication between tumor cells and endothelial cells, and exosomes from tumor cells contain protein, mRNAs and miRNAs, that promote angiogenesis of endothelial cells (60). Wang et al. indicated that exosomes isolated from VEGFC-treated adipose-derived stem cells promoted proliferation, migration, and tube formation of lymphatic endothelial cells (61). Hosseini et al. found that curcumin could inhibit the angiogenesis of HUVECs by regulation of FAK/p38 MAPK pathway (16). In this study, we found that HHT combined with curcumin treatment remarkedly reduced VEGF levels in exosomes derived from Raji cells. Interestingly, these exosomes with downregulated VEGFA notably inhibited proliferation, migration and angiogenesis of HUVECs. Moreover, application of Raji/combination exosomes to HUVECs significantly decreased the expression of VEGF and the phosphorylation of Akt, suggesting that VEGF/Akt signaling is inactivated by Raji/combination exosomes in HUVECs. Meanwhile, our findings were supported by the findings reported by Sedremomtaz et al., which indicated that inhibition of the Akt signaling pathway can suppress tumor cell proliferation, migration and tube formation (62). Lamanuzzi et al. found that activation of mTOR/PI3K/Akt pathway exhibits an important role in promoting cancer cell invasion and angiogenesis (63, 64). These data indicated that application of exosomes with downregulated VEGFA could inhibit lymphoma cell growth and angiogenesis *in vitro via* inactivation of VEGF/Akt signaling pathway. Furthermore, we found that Raji/combination exosomes could reduce the tumor volume and weight of Raji transplanted tumor *in vivo*. Although combination treatment exhibited anti-tumor effects in Raji cells *in vitro* and *in vivo*, the therapeutic potentialities of curcumin combined with HHT in lymphoma should be explored in both pre-clinical and clinical studies in the future.

Evidence has shown that delivering the anti-tumor drugs to tumors using engineered exosomes have potential value for clinical applications (65, 66). Zibaei et al. found that curcumin could be loaded into exosomes by a single-step nano-precipitation method, and then exhibited potent tumoricidal effects on colorectal cancer cells (67). Sun et al. showed that exosomes can be used drug delivery vehicles to deliver curcumin to target cells, thus improving the anti-inflammatory activity of curcumin (68). Thus, in the future, we aimed to investigate whether exosome could deliver curcumin and HHT to lymphoma cells, and then exerted tumoricidal effects.

In this study, we found that combination treatment exhibited anti-tumor effects in Raji cells through downregulation of VEGF/AKT signaling; however, these changes were reversed in the presence of VEGF. Thus, we speculated that VEGF-targeted therapy might be a treatment method for lymphoma. Evidences have shown that therapeutic monoclonal antibody (anti-CD20) has been approved for treating B-cell malignancies (4, 69). However, the role of combination of anti-CD20 antibody with other targeted therapies on lymphoma cells remains unclear. Thus, further study is needed to investigate the effect of combination of anti-CD20 antibody and anti-VEGF antibody on the proliferation and angiogenesis of lymphoma cells.

## Conclusion

In conclusion, combination of curcumin with HHT treatment displayed anti-lymphoma effect in Raji cells *via* inactivating VEGF/Akt and activating JNK signaling pathways. Moreover, Raji/combination exosomes could inhibit proliferation and angiogenesis of HUVECs *via* inactivation of VEGF/Akt signaling pathway. Therefore, curcumin combined with HHT might be considered as a therapeutic approach for the treatment of lymphomas.

## Data Availability Statement

The raw data supporting the conclusions of this article will be made available by the authors, without undue reservation.

## Ethics Statement

The animal study was reviewed and approved by First Affiliated Hospital of Zhejiang Chinese Medical University.

## Author Contributions

YZhang made major contributions to the conception, design and manuscript drafting of this study. JX, NZ, HG, XS, SD, JC, LY, and YZhou were responsible for data acquisition, data analysis, data interpretation, and manuscript revision. JS made substantial contributions to conception and design of the study and revised the manuscript critically for important intellectual content. All authors contributed to the article and approved the submitted version.

## Funding

1. Zhejiang Provincial Natural Science Foundation (No. Y19H270018, LY15H29004).
2. Special project for the modernization of traditional Chinese medicine in Zhejiang province (No. 2020ZX007).
3. National TCM clinical research base construction project (No. 2015H0105).
4. National Natural Science Foundation of China (No. 81800138).

## Conflict of Interest

The authors declare that the research was conducted in the absence of any commercial or financial relationships that could be construed as a potential conflict of interest.

## Publisher’s Note

All claims expressed in this article are solely those of the authors and do not necessarily represent those of their affiliated organizations, or those of the publisher, the editors and the reviewers. Any product that may be evaluated in this article, or claim that may be made by its manufacturer, is not guaranteed or endorsed by the publisher.

## Supplementary Material

Supplementary Figure 1 Combination of curcumin with HHT inhibited the viability and migration of lymphoma cells *via* downregulation of VEGFA. **(A)** RT-qPCR analysis of VEGFA level in Raji cells transfected with VEGFA siRNA2 and VEGFA siRNA2. **(B)** Raji cells were treated with 5 ng/mL HHT and 10 μM curcumin or treated with HHT, curcumin and VEGFA siRNAs. CCK-8 assay was used to detect cell viability. **(C)** Cell migration was detected by transwell migration assay. **P < 0.01 compared with control group; ^##^P < 0.01 compared with HHT + curcumin group.

Supplementary Figure 2 Combination of curcumin with HHT inhibited VEGF secretion by Raji cells *via* inactivation of the transcription factors STAT3 and SP1. Raji cells were treated with 5 ng/mL HHT or/and 10 μM curcumin for 72 h or treated with HHT, curcumin and VEGF. **(A, B)** Expression levels of p-STAT3, STAT3 and SP1 in Raji cells were detected with western blotting. The relative expressions of p-STAT3 and SP1 in cells were quantified *via* normalization to STST3 and β-actin. *P < 0.05, **P < 0.01 compared with control group; ^##^P < 0.01 compared with HHT group; ^^^^P < 0.01 compared with HHT + curcumin group.

Supplementary Figure 3 Combination of curcumin with HHT upregulated the levels of miR-150, miR-22, miR-206 and miR-34a in Raji cells. RT-qPCR analysis of miR-150, miR-22, miR-206 and miR-34a levels in Raji cells treated with 5 ng/mL HHT and 10 μM curcumin. **P < 0.01 compared with control group.

Supplementary Figure 4 Interactions with the microenvironment could protect Raji cells from combination treatment-induced apoptosis. resistance. Raji cells were co-cultured on a confluent monolayer of HS-5 stromal cells for 24 h. Then, Raji cells were treated with 5 ng/mL HHT and 10 μM curcumin for another 72 h. **(A)** CCK-8 assay was used to detect cell viability. **(B)** Flow cytometry assay was used to detect cell apoptosis. **P < 0.01 compared with control group; ^##^P < 0.01 compared with Plastic group.

## Abbreviations

A. lumbricoides, Ascaris lumbricoides; A. suum, Ascaris suum A. viteae, Acanthocheilonema viteae; Ag85A, Antigen 85A; Ag85B, Antigen 85B; AHR, Airway hyperreactivity; AIP-2, anti-inflammatory protein-2; APC, Antigen presenting cell; BCG, Bacille Calmette-Guérin; DCs, Dendritic cells; E. vermicularis, Entrobius vermicularis; ES, Excretory/secretory protein; H. polygyrus, Heligosomoides polygyrus; i.d., Intradermal; i.n., Intranasal; IL, Interleukin; ISAAC, International Study of Asthma and Allergies in Childhood; M. bovis, Mycobacterium bovis; Mtb, Mycobacterium tuberculosis bacteria; N. americanus, Necator americanus; N. brasiliensis, Nippostrongylus brasiliensis; OVA, Ovalbumin; RD, Region of difference; S. japonicum, Schistosoma japonicum; S. mansoni, Schistosoma mansoni; S. stercoralis, Strongyloides stercoralis; Smteg, schistosomula tegument; T. canis, Toxocara canis; T. spiralis, Trichinella spiralis; T. trichiura, Trichuris trichiura; TB, Tuberculosis; TGF, Transforming growth factor; Th, T helper; TLR, Toll-like receptor; TNF, Tumor necrosis factor; TP, Total protein; Tregs, T regulatory cells.
