# Supplementary figures and images for: Curcumin in Combination With Omacetaxine Suppress Lymphoma Cell Growth, Migration, Invasion, and Angiogenesis via Inhibition of VEGF/Akt Signaling Pathway
Source: Front Oncol. 2021 Aug 11;11:656045. doi: 10.3389/fonc.2021.656045 (PMC8386016; doi:10.3389/fonc.2021.656045)

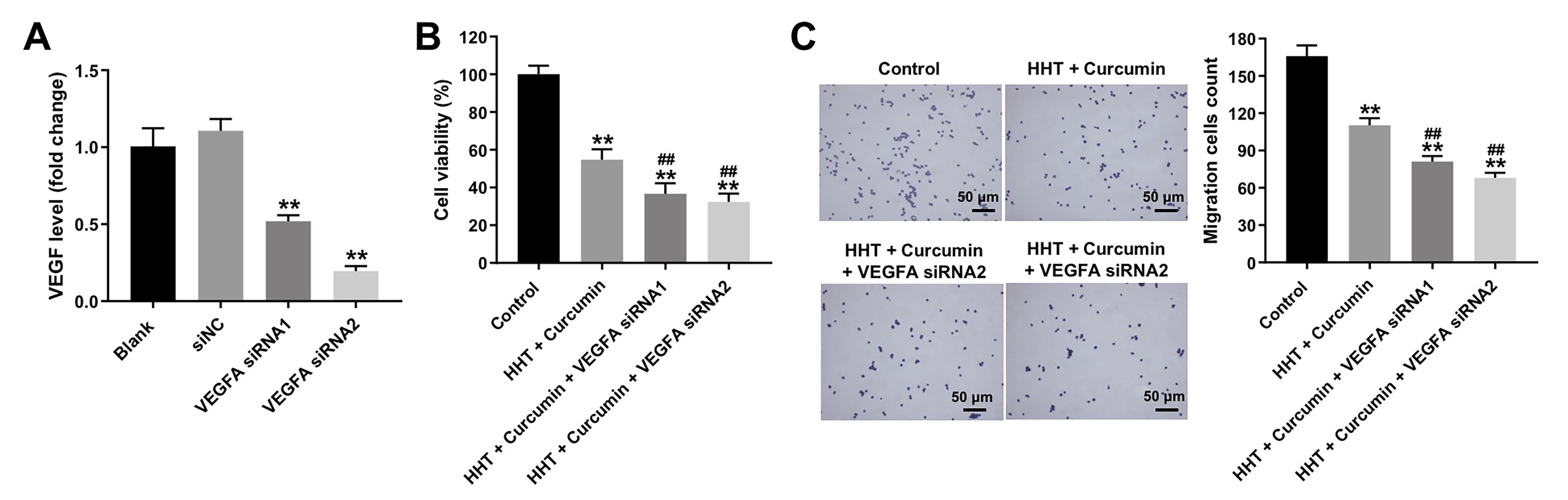

Supplement: Supplementary Figure 1 — Combination of curcumin with HHT inhibited the viability and migration of lymphoma cells via downregulation of VEGFA. (A) RT-qPCR analysis of VEGFA level in Raji cells transfected with VEGFA siRNA2 and VEGFA siRNA2. (B) Raji cells were treated with 5 ng/mL HHT and 10 μM curcumin or treated with HHT, curcumin and VEGFA siRNAs. CCK-8 assay was used to detect cell viability. (C) Cell migration was detected by transwell migration assay. **P < 0.01 compared with control group; ##P < 0.01 compared with HHT + curcumin group. [file Image_1.jpeg]

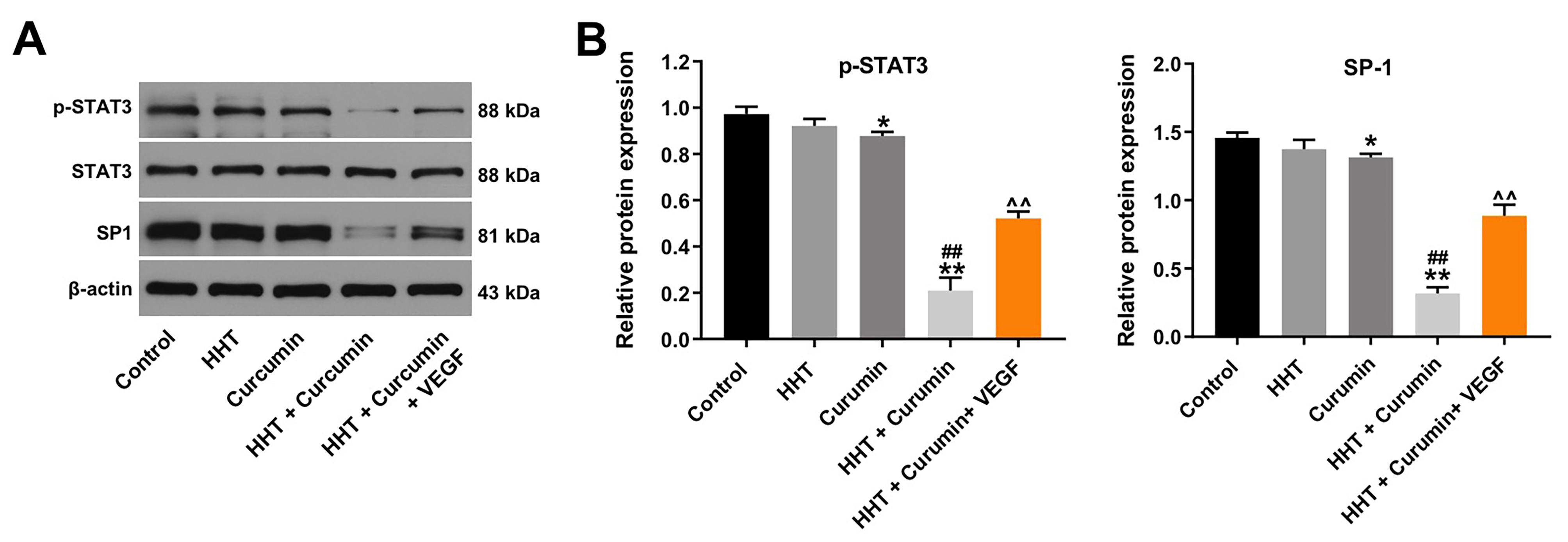

Supplement: Supplementary Figure 2 — Combination of curcumin with HHT inhibited VEGF secretion by Raji cells via inactivation of the transcription factors STAT3 and SP1. Raji cells were treated with 5 ng/mL HHT or/and 10 μM curcumin for 72 h or treated with HHT, curcumin and VEGF. (A, B) Expression levels of p-STAT3, STAT3 and SP1 in Raji cells were detected with western blotting. The relative expressions of p-STAT3 and SP1 in cells were quantified via normalization to STST3 and β-actin. *P < 0.05, **P < 0.01 compared with control group; ##P < 0.01 compared with HHT group; ^^P < 0.01 compared with HHT + curcumin group. [file Image_2.jpeg]

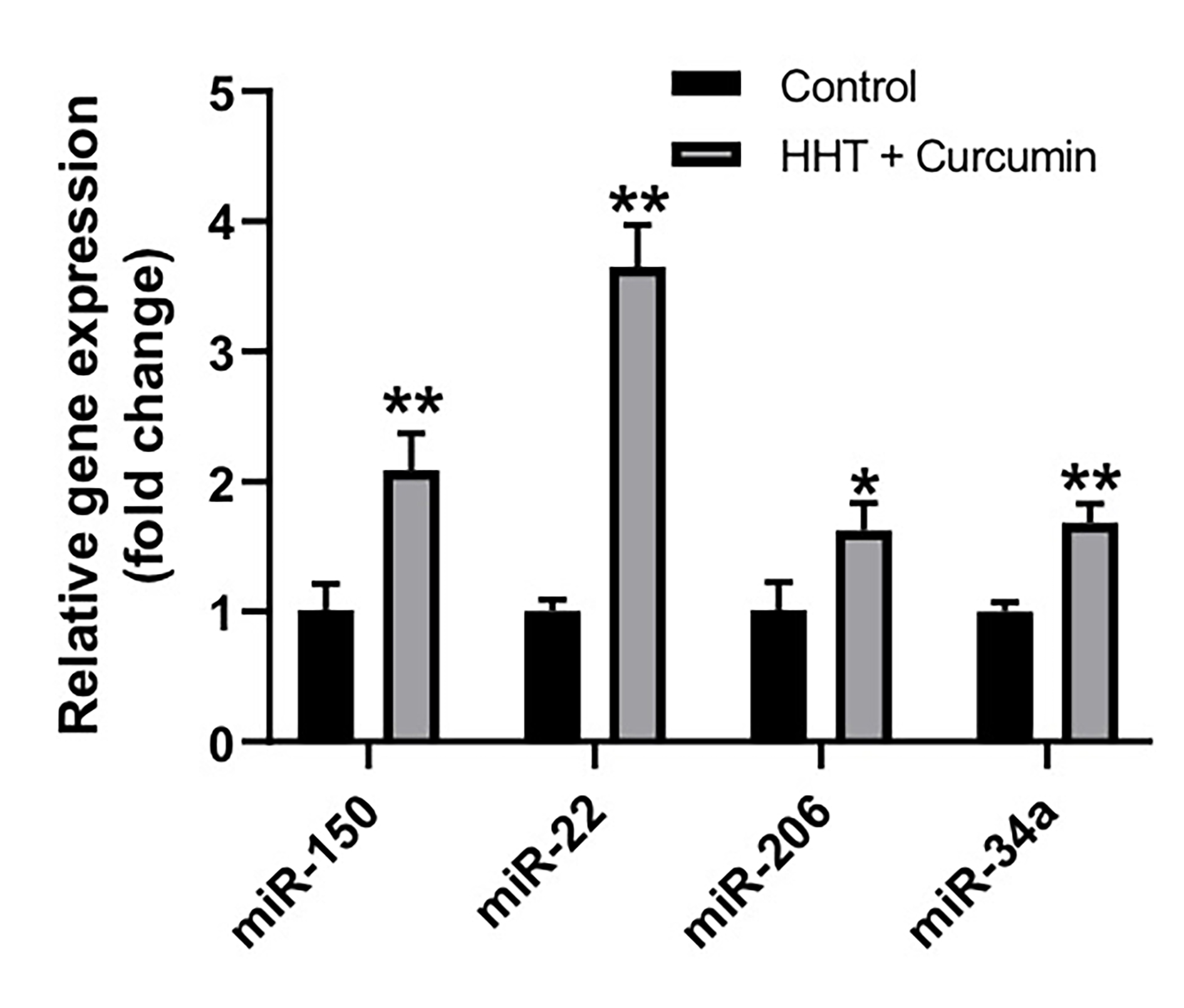

Supplement: Supplementary Figure 3 — Combination of curcumin with HHT upregulated the levels of miR-150, miR-22, miR-206 and miR-34a in Raji cells. RT-qPCR analysis of miR-150, miR-22, miR-206 and miR-34a levels in Raji cells treated with 5 ng/mL HHT and 10 μM curcumin. **P < 0.01 compared with control group. [file Image_3.jpeg]

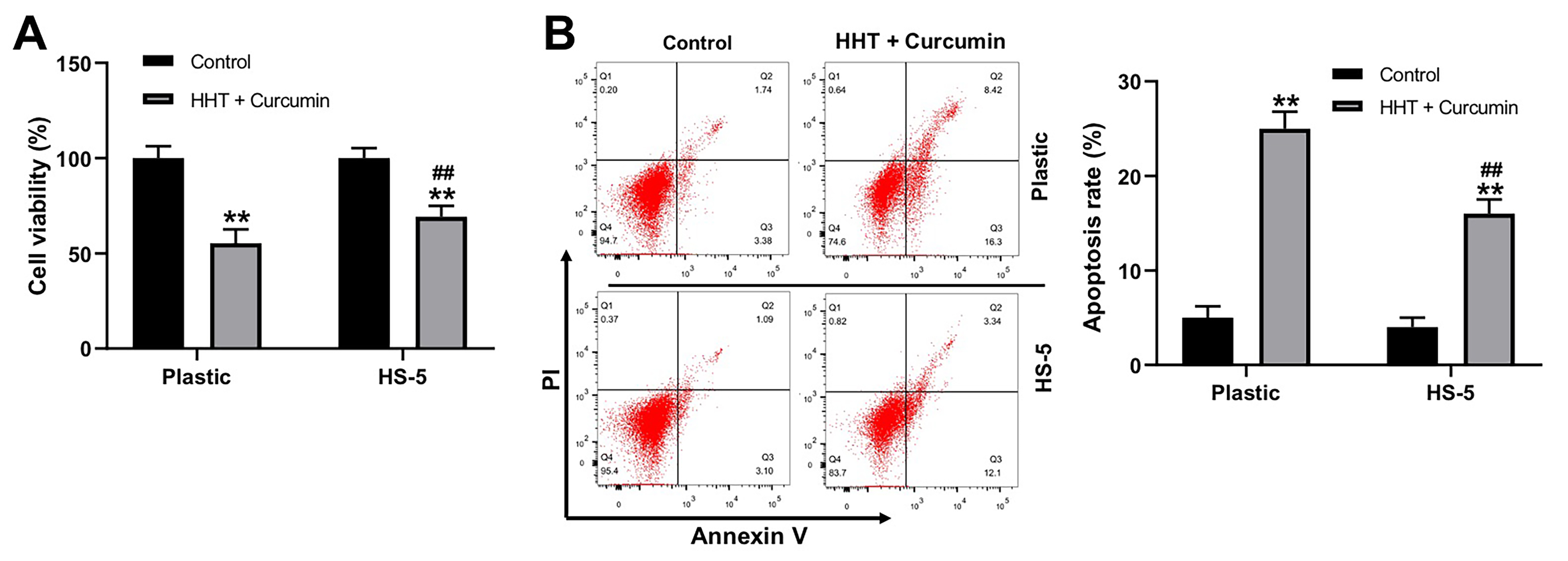

Supplement: Supplementary Figure 4 — Interactions with the microenvironment could protect Raji cells from combination treatment-induced apoptosis. resistance. Raji cells were co-cultured on a confluent monolayer of HS-5 stromal cells for 24 h. Then, Raji cells were treated with 5 ng/mL HHT and 10 μM curcumin for another 72 h. (A) CCK-8 assay was used to detect cell viability. (B) Flow cytometry assay was used to detect cell apoptosis. **P < 0.01 compared with control group; ##P < 0.01 compared with Plastic group. [file Image_4.jpeg]
